# Therapeutic Angiogenesis Using Basic Fibroblast Growth Factor in Combination with a Collagen Matrix in Chronic Hindlimb Ischemia

**DOI:** 10.1100/2012/652794

**Published:** 2012-05-15

**Authors:** Jianyin Zhou, Yilin Zhao, Jinling Wang, Sheng Zhang, Zhengjin Liu, Maochuan Zhen, Yun Liu, Pingguo Liu, Zhenyu Yin, Xiaomin Wang

**Affiliations:** ^1^Department of Hepatobiliary Surgery, Zhongshan Hospital, Xiamen University, Xiamen 361001, China; ^2^Department of Emergency, Zhongshan Hospital, Xiamen University, Xiamen 361001, China; ^3^Department of Pathology Zhongshan Hospital, Xiamen University, Xiamen 361001, China; ^4^Digestive Institute of Xiamen University, Xiamen 361001, China

## Abstract

Although therapeutic angiogenesis by angiogenic cytokines is a feasible strategy to improve regional blood flow in ischemic regions, the optimal delivery mode needs to be established. Here we designed a complex of collagen matrix (CM) and basic fibroblast growth factor (bFGF) and evaluated its proangiogenic effect in ischemic hindlimbs. The bFGF-CM was prepared using lyophilization. The morphology, porosity and toxicity of CM were examined. The bFGF releasing profile and bioactivity of released bFGF were assessed. bFGF-CM was intramuscularly implanted into the rabbit ischemic hindlimb model. Oxygen saturation parameters (OSP) of ischemic hindlimbs was measured to evaluate the extremity perfusion at intervals. Histological examination was performed to evaluate the level of angiogenesis. The CM and bFGF-CM were of identical multiporous structure lacking cytotoxicity. The releasing profile lasted 10 days and the released bFGF remained bioactive. OSP in bFGF-CM group was significantly higher than that in CM, bFGF and ischemic groups at 2 and 4 weeks. The number of capillaries and mature vessels in bFGF-CM group were significantly greater than that in untreated control, CM and bFGF groups. Therefore, bFGF-CM enables the safe and effective long-term release of bFGF with improved angiogenesis in ischemic hindlimbs compared with CM devoid of bFGF.

## 1. Introduction

Regeneration therapy with angiogenic cytokines has shown promising results for chronic ischemic hindlimb [1]. Among the cytokines used, basic fibroblast growth factor (bFGF) has been shown to be a safe and potentially effective therapy to reduce lower extremity ischemia either on its own or in conjunction with bypass surgery or percutaneous catheter-based recanalization [2, 3]. However, the optimal mode of delivery of the growth factor remains to be established.

Current angiogenic cytokine therapies to regenerate ischemic tissues in the body primarily rely on the bolus administration of the growth factor or direct injection into the blood stream [3]. The results of existing studies are not consistent [4, 5]. The bioactivity of bFGF could rapidly disappear in solution form or the growth factors could be rapidly cleared from the tissue [6]. Preclinical trials of direct administration of angiogenic cytokines have shown some limitations, whereby bFGF stimulated vessel growth for a transient period but resulted in fragile and leaky vessels that were prone to regression. Only the formation of mature blood vessels can bring about substantial tissue function and perfusion enhancement [7].

Thus, the exact mode of administration of growth factors for improving ischemic tissue function and perfusion remains uncertain and needs further exploration. Previous results suggest that a long-term controlled release system is required. In order to explore the most efficient mode of administration, sustained-release delivery systems using biodegradable materials into which the growth factor can be impregnated have been studied. As the biodegradable materials are degraded, growth factors are gradually released over time *in situ* [5, 8–10]. One approach involves the localized, sustained release of growth factors at the site of ischemia from biodegradable gelatin microspheres. These microspheres have been shown to be successful in protecting the cytokines from enzymatic degradation [11–15].

In this study, we developed a multiporous collagen matrix (CM) to impregnate the bFGF so as to prolong the release of bFGF from the matrix. We hypothesized that sustained release of bFGF over a long period of time could improve revascularization and reperfusion in a critical limb ischemic model.

## 2. Materials and Methods

### 2.1. Preparation of the CM and the bFGF-CM Complex

The type I bovine collagen (GIBCO Invitrogen Corp) suspension containing 2.5% collagen and heparin (50 U/mL, Shanghai First Biochemical and Pharmaceutical, China, 50 U/mL) was prepared. The collagen suspension was centrifuged to remove bubbles and was subsequently mixed gently into paste. To produce the complex of bFGF-CM, 15 *μ*g/mL of bFGF (Sigma) was added to the 2.5% collagen solution. The collagen solution alone or that containing bFGF was poured into a frame mold. The samples were frozen at −80°C and then lyophilized for 12 h to obtain a CM or bFGF-CM. The samples were sterilized with ethylene oxide gas at room temperature and stored under vacuum until use. The porosity of the matrix was measured by the 100% ethanol liquid replacement method. In order to maintain the bioactivity of bFGF, no crosslinking or other chemical agent was added. The collagen matrix was mounted on an aluminum stub, coated with gold using a Sputter Coater, and examined with a scanning electron microscope (SEM) (Tescan, Czech Republic).

### 2.2. Cytotoxicity of the CM

Rabbit bone-marrow-derived mesenchymal stromal cells (MSCs) were isolated, expanded, and used to test the cytotoxicity of the CM. The third passage MSCs were first seeded in a 96-well microplate at a density of 1.0 × 10^4^ cells. Each well contained 200 *μ*L Dulbecco's modified Eagle's medium (DMEM; Invitrogen) supplemented with 10% fetal bovine serum (FBS) and 1% penicillin-streptomycin solution at 37°C in 5% CO_2_ atmosphere. After 24 h of culture, the medium was replaced with fresh medium containing 200 *μ*g/mL of collagen particles, which had been scraped from the CM. The medium was replaced every 2 days and MSCs cultured in DMEM were considered as the control. The cell number was counted with a hemocytometer at specific intervals.

### 2.3. Rehydration Analysis

The dried CM, with a weight of 10 ± 0.1 g, was immersed in PBS, and after 24 h, samples were collected. Then, the samples were blotted with tissue to remove the liquid on the surface. The swelling ratios Rr (%) of test samples were calculated using the following equation: (*W*_*s*_ − *W*_*d*_)/  *W*_*d*_, where *W*_*s*_  is the weight of the swollen test sample and *W*_*d*_ is the weight of the dried test sample.

### 2.4. Sustained Release Analysis

The bFGF-CM complex was added to a tube that contained 10 mL PBS at 37°C and shaken at 40 rpm. At intervals, the PBS was collected for bFGF analysis and replaced with fresh PBS. bFGF in the collected solution was determined by the enzyme-linked immunosorbent assay, which was performed according to the manufacturer's instructions (Quantikine human bFGF immunoassay; R & D Systems, Minneapolis, MN, USA).

### 2.5. Bioactivity of the Released bFGF

This experiment was to evaluate the bioactivity of bFGF which was incorporated into collagen matrix. A total of 15 mg of bFGF-CM was cut into pieces, which were added to a centrifuge tube containing 15 mL of DMEM. The tube was then vibrated at 120 rpm at 37°C. After 1 day of releasing, the tube was centrifuged and the supernatant was used to analyze bFGF bioactivity. Rabbit bone-marrow-derived MSCs were isolated, purified, and seeded in a 96-well microplate at a density of 1 × 10^4^ cells per well containing 200 *μ*L of culture medium at 37°C in a 5% CO_2_ atmosphere. After 24 h, the medium was replaced with the release medium supplemented with 10% FBS and 1% penicillin-streptomycin solution. MSCs cultured in DMEM medium were considered as the control. The cell number was counted with a hemocytometer at day 2 and day 5.

### 2.6. Ischemic Hindlimb Model

All experiments that involved the use of animals were approved by the Institutional Animal Care and Use Committee at Xiamen University. New Zealand White rabbits were anesthetized with an intramuscular injection of ketamine (30 mg/kg) and an intravenous injection of pentobarbital (30 mg/kg) and ventilated with a mixture of oxygen, nitrogen, and isoflurane during the operation. A longitudinal incision was made on the right hindlimb and the femoral artery was removed. The incision was then sutured, all animals were regularly examined and treated with analgesic and antibiotic drugs for 5 days. The animals were used for experiments at 10 days after surgery.

### 2.7. Treatment of Ischemic Hindlimb

The ischemic hindlimb models were randomly divided into the ischemic, bFGF, CM, and bFGF-CM groups (*n* = 5). In the ischemic group, the animals did not receive any treatment. In the bFGF group, on average 15 *μ*g of bFGF in 3 mL of PBS was injected into two sites along the sides of the longitudinal incision, each site was 0.8 cm^2^ in area, and the injection was 1 cm in depth. In the CM group, the CM (0.8 cm in diameter) was implanted into the same sites as that in bFGF group. In the bFGF-CM group, the complex of bFGF and CM (0.8 cm in diameter) was implanted into the sites described previously. The implanted sites were stitched as a marker.

### 2.8. Oxygen Saturation Parameter Analysis

The hindlimbs of the animals were tested for hemoglobin oxygen saturation to evaluate the ischemic hindlimb perfusion at 1, 2, and 4 weeks. Oxygen saturation of hemoglobin was checked by the hemoglobin absorption spectrum with a spectrometer (GE Medical Systems Information Technologies) at a wavelength range from 500 to 620 nm by using a clamp probe placed on both of the animals' hind feet, the hair of which had been removed. Measurements were performed in a chamber at room temperature. The data was calculated by the parameter ratio of the right foot/left foot in each animal.

### 2.9. Histological and Immunohistochemical Examination

The animals were killed at 4 weeks. Samples from the implanted sites with stitched markers in ischemic muscle were treated with 10% neutralized formalin, embedded in paraffin, and cut into 4 *μ*m sections. The samples in the ischemic group without treatment were harvested from the same sites as the other groups. The sections were stained with hematoxylin and eosin. The specimens were also immunohistochemically stained with an antibody against von Willebrand factor (vWF; Dako, Carpinteria, CA, USA) to determine the quantitative capillary density and with an antismooth muscle *α*-actin (SMA) antibody (Dako) to evaluate the quantitative maturation of arteries. Positive staining indicated the presence of capillaries or mature vascular vessels. Vessel densities were calculated as the number of vessels/mm^2^.

### 2.10. Statistical Analysis

Data is expressed as means ± SD. Analysis of variance (ANOVA) followed by Student's test was used to determine the significant differences among the groups. All data were considered significant at *P* < 0.05.

## 3. Results

### 3.1. Characterization of the CM and the bFGF-CM Complex

**Figure 1:**
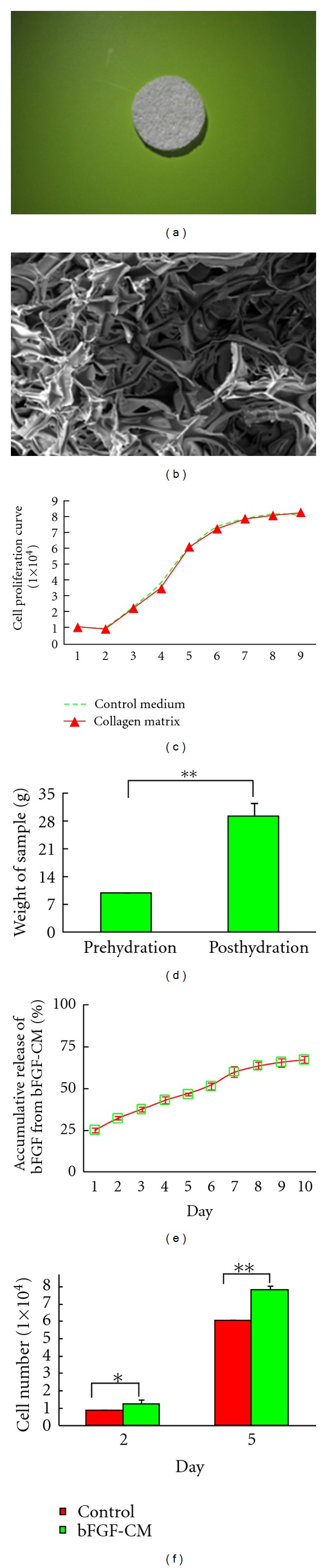
Characterization of the CM and the bFGF-CM complex. (a) Gross overview of the bFGF-CM complex. (b) Scanning electron microscopy indicated that bFGF-CM had a multiporous structure (bar = 100 *μ*m). (c) Proliferation assays showed that CM did not have a cytotoxic effect on MSCs. (d) Rehydration analysis of the CM. (e) The kinetic profile of bFGF release. (f) The bioactivity analysis of the bFGF released from the bFGF-CM complex (*P < 0.05, **P < 0.01).

Both the CM and the bFGF-CM complex were white multiporous collagen matrices that look the same from the scanning electron microscope image and the gross view (Figure 1(a)). The average pore size was 100 *μ*m (Figure 1(b)) with 80.2% porosity. The pores in the CM are interconnected (Figure 1(b)). The CM had a high rehydration capacity; the rehydration ratio reached 2.94 ± 3.4 (Figure 1(d)).

### 3.2. Cytoxicity of the CM

As shown in Figure 1(c), the proliferation rate of the MSCs cultured with CM particles was not significantly different from the control, indicating that CM did not affect the proliferation of MSCs. These findings indicated that the effect of toxicity of the crosslinking agent could be excluded in our study (Figure 1(c)).

### 3.3. bFGF Releasing Profile

The release of bFGF from the bFGF-CM complex was constant and sustained, and the cumulative release increased linearly with time (Figure 1(e)). By day 10 of the release study, 67.3% ± 2.2% of  bFGF was released to the external medium. The releasing profile reached the plateau phase by day 8.

### 3.4. Bioactivity of Released bFGF *In Vitro*

In order to determine whether the bFGF incorporated into CM was inactivated or not, the bioactivity of the released bFGF was established by assessing its ability to stimulate the expansion of MSCs *in vitro*. The results presented in Figure 1(f) reveal that MSCs cultured in the release media collected on days 2 and 5 showed increased proliferation *in vitro* compared to those cultured in control media.

### 3.5. Oxygen Saturation Analysis in Ischemic Hindlimbs

**Figure 2:**
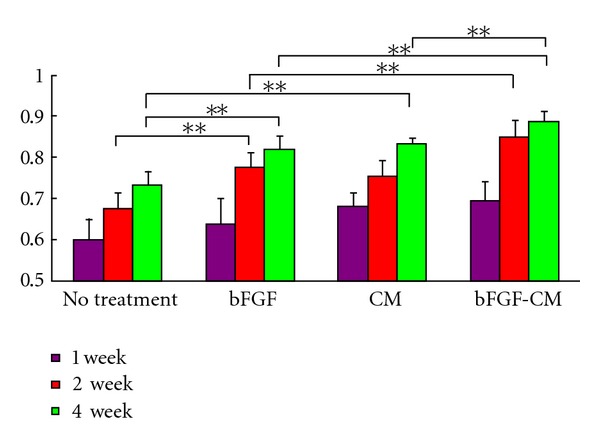
The oxygen saturation parameter ratio of the right, operated hindlimb/the left, nonoperated hindlimb at 1, 2, and 4 weeks (**P < 0.01).

The oxygen saturation of hemoglobin was measured at an equivalent position in hindlimbs that had been operated on and those that had not. After treatment, the pedal oxygen saturation (an indicator of extremity perfusion) was examined at 1, 2, and 4 weeks. In the bFGF-CM group, the oxygen saturation was 0.7 ± 0.05, 0.85 ± 0.04, and 0.89 ± 0.03 in weeks 1, 2, and 4, respectively. The parameters were 0.69 ± 0.02, 0.75 ± 0.04, and 0.83 ± 0.01 in the CM group and 0.64 ± 0.07, 0.78 ± 0.04, and 0.82 ± 0.04 in the bFGF group. In the ischemic group, the oxygen saturation measures were 0.6 ± 0.05, 0.68 ± 0.04, and 0.73 ± 0.03. In all groups, oxygen perfusion improved gradually with time. However, in the bFGF-CM group, the oxygen saturation parameters were significantly higher than those in the ischemic, bFGF, and CM groups at weeks 2 and 4 (*P* < 0.01). The oxygen saturation parameters in the bFGF and CM groups were significantly higher than those in the ischemic group at week 2 and week 4 (*P* < 0.01). The oxygen saturation parameters in the CM group were similar to those in the bFGF group at the different time intervals (*P* > 0.05) (Figure 2).

### 3.6. Gross View and Histological and Immunohistochemical Analysis

**Figure 3:**
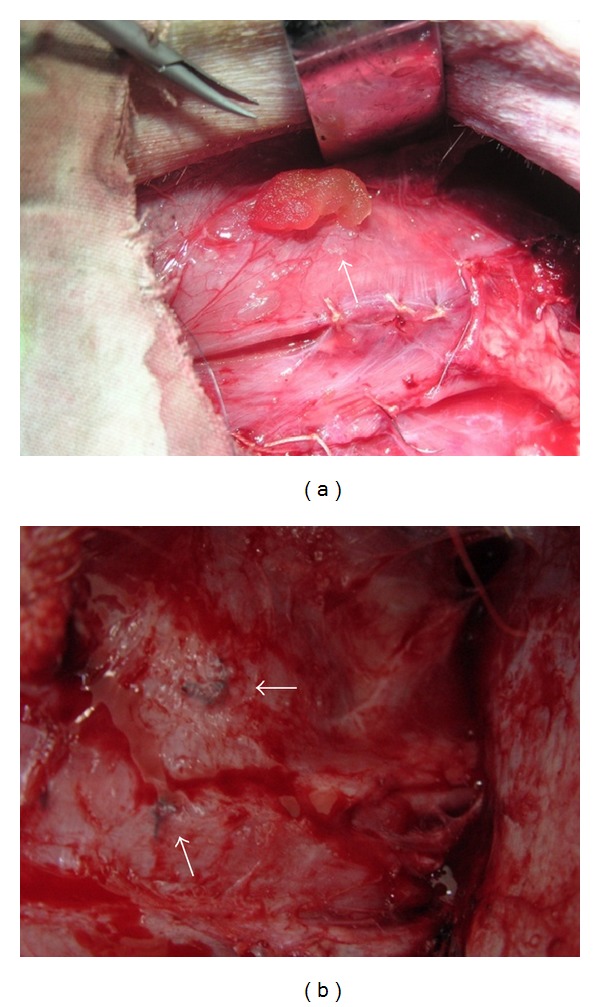
(a) bFGF-CM was being implanted into the ischemic hindlimb muscle, the implanted sites were stitched as markers for samples collection. (b) At harvest, ischemic hindlimb tissue regenerated well; the stitched markers indicated the bFGF-CM implantation sites.

**Figure 4:**
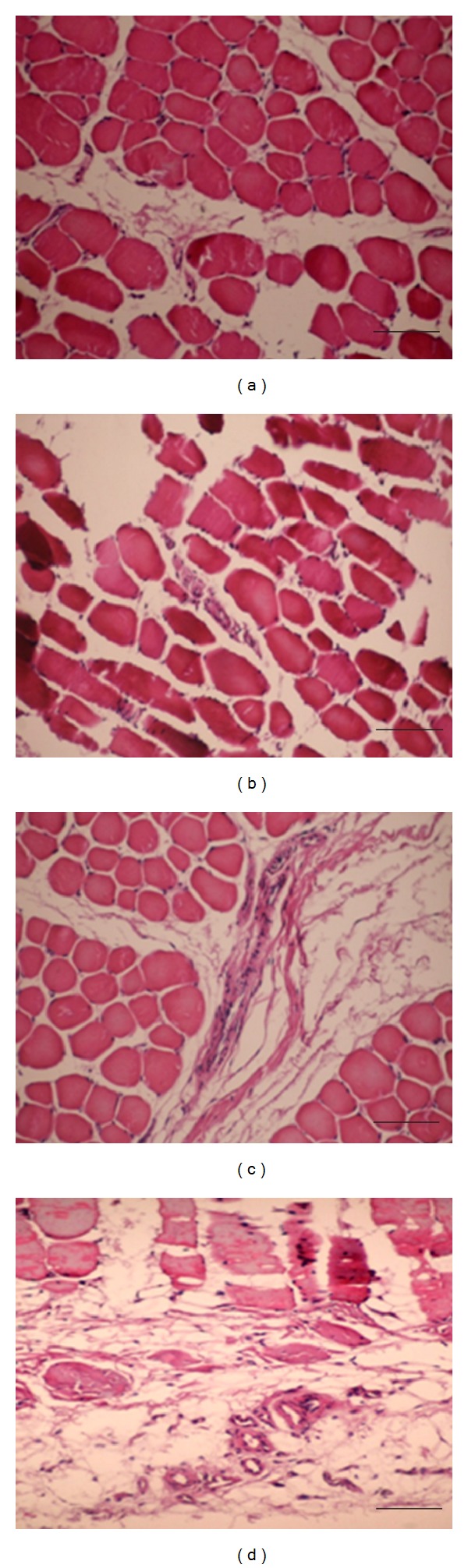
Hematoxylin-eosin analysis for revascularization in the muscles of the ischemic hindlimb 4 weeks after implantation. (a) No treatment; (b) bFGF; (c) CM; (d) bFGF-CM (bar = 50 *μ*m).

**Figure 5:**
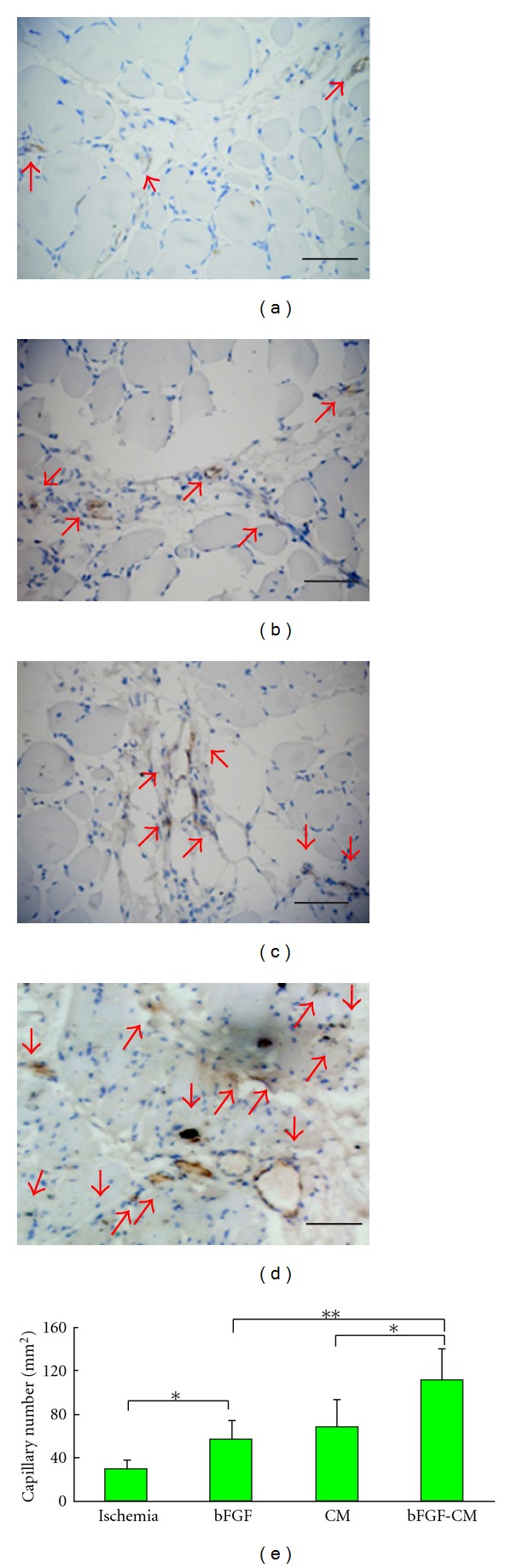
Immunohistochemical analysis for von Willebrand factor in ischemic hindlimbs 4 weeks after implantation. (a) No treatment; (b) bFGF; (c) CM; (d) bFGF-CM (bar = 50 *μ*m). (e) Histogram of the number of capillaries in the ischemic, bFGF, CM, and bFGF-CM groups (*P < 0.05, **P < 0.01).

**Figure 6:**
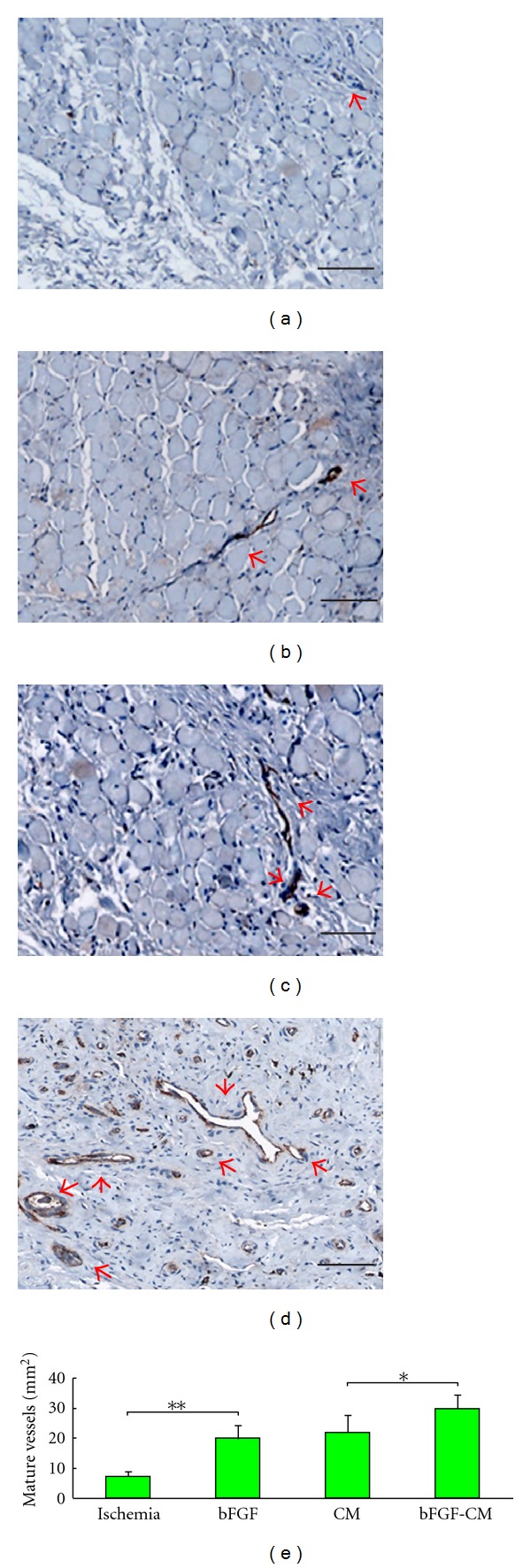
Immunohistochemical analysis for smooth muscle *α*-actin in ischemic hindlimbs 4 weeks after implantation. (a) No treatment; (b) bFGF; (c) CM; (d) bFGF-CM (bar = 100 *μ*m). (e) Histogram of the number of mature vessels in the ischemic, bFGF, CM, and bFGF-CM groups (*P < 0.05, **P < 0.01).

The bFGF-CM complex was implanted intramuscularly *(Figure 3(a) showed that the bFGF-CM was being implanted into the ischemic muscle). *After 4 weeks, the ischemic tissue regenerated well (Figure 3(b)). More vessels formed in the bFGF-CM group than in the CM, bFGF, and ischemic groups (Figure 4). The densities (indicated as vessels/mm^2^) of capillaries with positive staining for vWF were 111.2 ± 28.9, 67.8 ± 25.9, 56.6 ± 17.5, and 29.4 ± 8.2 in the bFGF-CM, CM, bFGF, and ischemic groups, respectively. The capillary number in the bFGF-CM group was significantly more than that in the CM and bFGF groups (*P* < 0.05). The number of capillaries in the CM and bFGF groups was significantly higher than that in the ischemic control group (*P* < 0.05). The difference in capillary number between the CM and bFGF groups was not significant (Figure 5). The number of mature vessels with positive staining for SMA was significantly more in the bFGF-CM group (Figure 6(d)) than that in the CM (Figure 6(c)), bFGF (Figure 6(b)), and ischemic groups (Figure 6(a)). The density of mature vessels in the bFGF-CM group was 30 ± 4.8 vessels/mm^2^, which was significantly higher than that in the CM (22.2 ± 5.7 vessels/mm^2^), bFGF (20 ± 4.7 vessels/mm^2^), and ischemic groups (7.4 ± 1.5 vessels/mm^2^; *P* < 0.05; Figure 6(e)). The number of mature vessels in the CM and bFGF groups was significantly higher than that in the control ischemic group (*P* < 0.05). There was no significant difference between the CM and bFGF groups (Figure 6).

## 4. Discussion

In the present study, we demonstrated that spatial and temporal sustained delivery of bFGF from a CM in a rabbit model of critical hindlimb ischemia resulted in neovessel formation and an increase in hindlimb perfusion. The release of bFGF from the bFGF-CM complex was sustained for 10 days. Additionally, the bFGF incorporated into CM remained bioactive as demonstrated by the ability to stimulate MSC proliferation *in vitro*. The oxygen saturation in the bFGF-CM group was significantly higher than that in the CM, bFGF and ischemic groups 2 weeks after the bFGF-CM complex was implanted. The extent of angiogenesis was determined by the amount of capillaries and mature vessels that were positive for antibodies against vWF and SMA, respectively. The number of capillaries or mature vessels was significantly higher in the bFGF-CM group than that in the CM, bFGF, and ischemic groups at week 4.

Regeneration therapy using angiogenic cytokines has shown promising results for chronic peripheral artery disease [16, 17]. Among these cytokines, bFGF is one of the most potent mitogens for therapeutic angiogenesis [18]. As chronic ischemic hindlimb needs long-term treatment, a sustained drug delivery system is urgently needed. The duration of exposure to bFGF may be a critical determinant of formation of stable neovasculature [19].

Biosynthetic matrices with bioactive and bioresorbable characteristics are becoming more important than ever before [20, 21]. Future biosynthetic matrices should possess both bioactive and bioresorbable properties to facilitate tissue regeneration and enhance the replacement of the matrix itself [22, 23]. Our previous report confirmed that gelatin microspheres loaded with bFGF improved capillary formation in the lower extremity compared with bFGF alone [11]. Therefore in the present study, we used another common biodegradable material, that is, collagen.

According to previous studies, the collagen biomaterial has prominent properties: firstly, the pore and porosity of the collagen matrix could be controlled [24]; secondly, cytokines could easily be impregnated into the collagen matrix without affecting its bioactivity [25]; thirdly, a stable and long-term sustained release of cytokines from the collagen matrix could be achieved [25]. Another reason for using a collagen matrix is based on our prior work in which we demonstrated the utility of collagen biomaterial for cell attachment and proliferation [26]. Moreover, collagen scaffold seeded with bone marrow-derived MSCs was a stronger angiogenic inducer than MSCs alone [27]. Collagen can induce proangiogenesis and provide long-term activation of gelatinase A, which is likely to be relevant to endothelial cell invasion during angiogenesis [28]. Our results were consistent with these studies.

The bFGF was released from the CM over a period of time and triggered therapeutically significant angiogenesis, including improved hindlimb perfusion and the formation of capillaries and mature blood vessels. These results suggest that angiogenesis depends on the sustained release of bFGF.

The study lasted for 4 weeks in order to decrease the formation of leaky vessels and facilitate the formation of pericytes or smooth muscle cells indicating the stable vasculature around endothelial cells [29]. In the bFGF-CM group, the oxygen saturation, capillary growth, and mature vessel formation were significantly more than those in the CM, bFGF, and ischemic groups. The percentages of the mature vessels/the total vessels in bFGF-CM, CM, bFGF, and ischemic groups were 24.8%, 29.8%, 33.3%, and 23.7%, respectively. There was more capillaries formation than mature vessels formation at 4 weeks. We speculated that if we prolonged the research, more capillaries would become mature vessels due to the bFGF sustained releasing profile. The angiogenesis improved the blood flow in ischemic hindlimbs. This improvement could be due to the release of bFGF at the ischemic site at the correct time for facilitation of therapeutic angiogenesis. Sustained release of bFGF may facilitate endothelial cell, smooth muscle cell, or pericyte recruitment resulting in the formation of stable, mature vasculature [30, 31].

The localized, timely, and sustained delivery of the growth factor used in this study enabled the control of therapeutic angiogenesis [32]. Additionally, using the system developed in this study we demonstrated that controlled delivery of bFGF could result in an increased growth and maturation of vessels as shown by immunohistochemical staining. Previous studies using bolus injections of growth factors had minimal success in sustaining mature vessel density and hindlimb blood flow because of the short half-life of common angiogenic growth factors [2]. The mature vessels induced by sustained delivery did not appear to regress, as indicated by the number of vessels [33].

In future clinical applications, doctors have to carefully consider the sites and ways of using the bFGF-CM complex for transplantation in patients with ischemic hindlimbs disorders. There are some differences between human beings suffering from ischemic hindlimb disorders and ischemic hindlimb animal models. In the human body, the arteries are narrowed or occluded, but they are present. However, in animal models, the arteries are removed. Therefore, according to our experience, the bFGF-CM should be implanted along the sides of the occluded artery to stimulate angiogenesis, which could connect the proximal and distal parts of the occluded artery and allow more blood supply and perfusion into the extremity. The ways of application of the bFGF-CM could be adjusted according to the condition; the bFGF-CM complex could be used in small pieces via injection or implanted in bulk (e.g., as a disc, similar to what we used in this study (Figure 3) during a surgery.

In conclusion, a complex composed of bFGF and multiporous CM was fabricated. This system showed no toxicity and ideal biocompatibility both *in vivo* and *in vitro*. Once implanted, the bFGF-CM complex showed higher efficacy in improving lower extremity perfusion, capillary density, and mature vasculature formation compared with the control groups in the chronic ischemic hindlimb model.
